# Systemic Optimization of Legume Nodulation: A Shoot-Derived Regulator, miR2111

**DOI:** 10.3389/fpls.2021.682486

**Published:** 2021-07-15

**Authors:** Nao Okuma, Masayoshi Kawaguchi

**Affiliations:** ^1^Division of Symbiotic Systems, National Institute for Basic Biology, Okazaki, Japan; ^2^Department of Basic Biology, School of Life Sciences, The Graduate University for Advanced Studies, SOKENDAI, Okazaki, Japan

**Keywords:** legume nodulation, autoregulation of nodulation, long-distance signaling, miR2111, C-terminally encoded peptide, CLAVATA3/EMBRYO-SURROUNDING REGION peptide, leucine-rich repeat receptor-like kinase, TOO MUCH LOVE

## Abstract

Long-distance signaling between the shoot and roots of land plants plays a crucial role in ensuring their growth and development in a fluctuating environment, such as with soil nutrient deficiencies. MicroRNAs (miRNAs) are considered to contribute to such environmental adaptation *via* long-distance signaling since several miRNAs are transported between the shoot and roots in response to various soil nutrient changes. Leguminous plants adopt a shoot-mediated long-distance signaling system to maintain their mutualism with symbiotic nitrogen-fixing rhizobia by optimizing the number of symbiotic organs and root nodules. Recently, the involvement and importance of shoot-derived miR2111 in regulating nodule numbers have become evident. Shoot-derived miR2111 can systemically enhance rhizobial infection, and its accumulation is quickly suppressed in response to rhizobial inoculation and high-concentration nitrate application. In this mini-review, we briefly summarize the recent progress on the systemic optimization of nodulation in response to external environments, with a focus on systemic regulation *via* miR2111.

## Introduction

Long-distance communication between the shoot and roots of land plants is crucial for coordinating growth and development in fluctuating environments such as soil nutrient deprivation at the whole-plant level (Ko and Helariutta, 2017). Phloem sap contains various microRNAs (miRNAs), and their accumulation is regulated by limitations the nutrients available to the plants, implying that shoot-to-root mobile miRNAs might be critical for adaptation to nutritional fluctuations (Pant et al., 2008, 2009; Buhtz et al., 2010). However, the molecular functions of such mobile miRNAs are largely unknown (Pagliarani and Gambino, 2019). Legumes maintain symbiosis with nitrogen-fixing bacteria, known as the rhizobia, in their roots through shoot-mediated long-distance signaling systems (Caetano-Anollés and Gresshoff, 1991; Ferguson et al., 2019; Gautrat et al., 2020b). Recently, miR2111 in legumes has attracted attention as a shoot-derived regulator of rhizobial infection that is crucial for symbiotic benefits.

Symbiotic interactions between legumes and rhizobia can be achieved by the formation of a host postembryonic root organ, called the nodule (Hirsch, 1992; Suzaki et al., 2015). Rhizobia can inhabit the nodules and fix atmospheric dinitrogen gas to produce ammonium ions, a plant-available form of nitrogen. To maintain this symbiosis, host plants must use photosynthates as energy for nodule formation and bacterial maintenance. Although this symbiotic interaction is beneficial under nitrogen-limiting conditions, the cost for these photosynthates is high. Thus, host plants must balance the benefits of nitrogen nutrition provided by rhizobia and the costs of carbon sources for nodulation. To optimize this symbiosis, legumes control nodule numbers using long-distance negative/positive feedback systems in response to rhizobial infection and soil nutrient availability (Caetano-Anollés and Gresshoff, 1991; Ferguson et al., 2019). Accumulating evidence suggests that shoot-derived miR2111 might play a central role in controlling nodule numbers (Tsikou et al., 2018; Gautrat et al., 2020a; Okuma et al., 2020; Zhang et al., 2021). Here, we summarize recent advances in the study of systemic regulation of nodulation in response to external environmental stimuli, particularly focusing on the relevance of shoot-derived miR2111.

## Molecular Basis of Autoregulation of Nodulation

**Figure 1:**
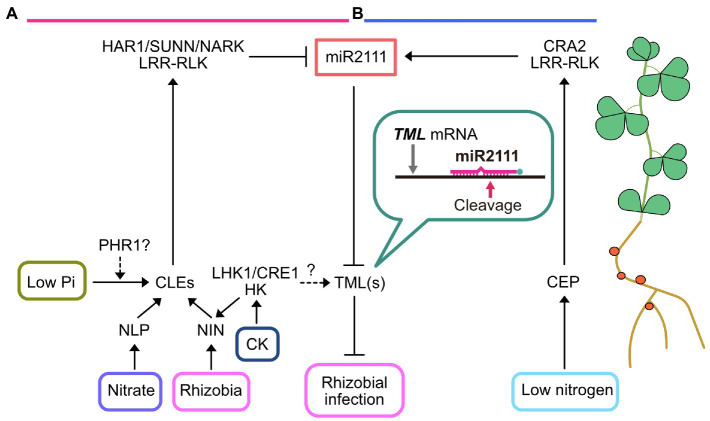
Current model of miR2111-mediated control of rhizobial infection. **(A)** Rhizobial infection and nitrate treatments induce CLAVATA3/EMBRYO-SURROUNDING REGION (CLE) small peptide production in roots. The expression of rhizobia- and nitrate-responsive specific CLE peptides is activated by transcription factors NODULE INCEPTION (NIN) and NIN-LIKE PROTEIN (NLP), respectively. Cytokinin (CK) application induces *CLE* expression depending on NIN through cytokinin receptor histidine kinases (HKs), such as LOTUS HISTIDINE KINASE1 (LHK1) and CYTOKININ RESPONSE1 (CRE1). Grafting experiments using *lhk1*-mutants demonstrated that the induction of these CLEs might partially depend on HK in the roots. It is also possible that LHK1 locally influences TOO MUCH LOVE (*TML*) expression in roots, which is independent of CLEs (dashed line with question mark). Moreover, PHOSPHATE STARVATION RESPONSE 1 (PHR1) likely contributes CLE expression in response to low-phosphate conditions. Arabinosylated derivatives of these CLE peptides are transported through xylem and directly recognized by leucine-rich repeat receptor-like kinases (LRR-RLKs), such as HYPERNODULATION ABERRANT ROOT1 (HAR1), SUPER NUMERIC NODULES (SUNN), and NODULE AUTOREGULATION RECEPTOR KINASE (NARK). These LRR-RLKs inhibit miR2111 accumulation in the shoot and systemically increase *TML* mRNA levels in the roots to inhibit rhizobial infection. **(B)** In contrast to autoregulation of nodulation (AON), CEP peptide is produced in the roots to systemically enhance rhizobial infection in response to low nitrogen availability. Root-derived CEP peptides are perceived by shoot-acting compact root architecture 2 (CRA2) LRR-RLK. CRA2 increases miR2111 levels and consequently enhances rhizobial infection in the roots. Hence, these two antagonistic regulatory events might maintain ideal nodule numbers to adapt to fluctuating nitrogen availability.

As an important strategy for maintaining mutualism with rhizobia, the host plant can implement a mechanism referred to as autoregulation of nodulation (AON) to control nodule numbers in conditions with sufficient rhizobia infection or ample amounts of available nitrate in the soil (Figure 1). Mutants that form excessive nodules (hypernodulation) due to a defect in AON are strongly inhibited in their growth, indicating that AON is important for host plants to gain benefits from this symbiotic interaction (Wopereis et al., 2000; Nishimura et al., 2002; Searle et al., 2003; Schnabel et al., 2005; Magori et al., 2009; Takahara et al., 2013). Moreover, the molecular basis of AON is widely conserved in legumes, such as *Lotus japonicus*, *Medicago truncatula*, *Glycine max* (soybean), and *Pisum sativum* (Figure 1; Ferguson et al., 2019). The initial step in AON is the induction of root-derived mobile signals, CLAVATA3/EMBRYO-SURROUNDING REGION (CLE)-related small peptides, designated as CLE ROOT SIGNAL1, 2, and 3 (CLE-RS1, CLE-RS2, and CLE-RS3) in *L. japonicus* (Okamoto et al., 2009, 2013; Nishida et al., 2016), CLE12 and CLE13 in *M. trunctula* (Mortier et al., 2010), and RHIZOBIA-INDUCED CLE1 (RIC1) and RIC2 in soybean (Reid et al., 2011, 2013), in response to rhizobial infection or high soil nitrate concentrations. The induction of genes encoding these CLE peptides is mediated by an RWP-RK-containing transcription factor, NODULE INCEPTION (NIN; Soyano et al., 2014; Laffont et al., 2020). Next, these CLE peptides are translocated into the shoot through xylem vessels and perceived by a leucine-rich repeat receptor-like kinase (LRR-RLK), such as HYPERNODULATION ABERRANT ROOT1 (HAR1) in *L. japonicus* (Wopereis et al., 2000; Krusell et al., 2002; Nishimura et al., 2002; Okamoto et al., 2013), SUPER NUMERIC NODULES (SUNN) in *M. truncatula* (Schnabel et al., 2005; Mortier et al., 2012), and NODULE AUTOREGULATION RECEPTOR KINASE (NARK) in soybean (Searle et al., 2003; Reid et al., 2011). Reciprocal grafting experiments demonstrated that these LRR-RLKs act in the shoot to systemically inhibit root nodule formation (Krusell et al., 2002; Nishimura et al., 2002; Searle et al., 2003; Schnabel et al., 2005). Arabinosylation of CLE peptides is necessary for direct recognition by LRR-RLKs (Okamoto et al., 2013; Imin et al., 2018; Hastwell et al., 2019). Putative genes encoding hydroxyproline-O-arabinosyl transferase, such as *ROOT DETERMINED NODULATION1* in *M. truncatula* and *PLENTY* in *L. japonicus*, has been identified and might be crucial for the part of the enzymatic reaction that produces arabinosylated derivatives of the CLE peptides (Kassaw et al., 2017; Yoro et al., 2019). In roots, TOO MUCH LOVE (TML), a Kelch repeat-containing F-box protein, inhibits nodulation downstream of LRR-RLKs (Magori et al., 2009; Takahara et al., 2013; Gautrat et al., 2019; Zhang et al., 2021).

The shoot-derived factors connecting LRR-RLKs, such as HAR1/SUNN/NARK, in the shoot and TML in the roots were unknown for a long time. Cytokinin was proposed as one such shoot-derived factor, since in *L. japonicus*, the expression of *ISOPENTENYL TRANSFERASE 3* (*IPT3*), which encodes a cytokinin biosynthetic enzyme, is induced in response to rhizobial infection in a HAR1-dependent manner (Sasaki et al., 2014). Indeed, cotyledon-feeding cytokinin can reduce nodule numbers. However, the mechanism underlying the inhibitory effect of cytokinin is unknown since cytokinin typically positively regulates nodule formation (Murray et al., 2007; Tirichine et al., 2007; Frugier et al., 2008). In soybean, the application of high concentrations of cytokinins *via* petioles decreases nodule numbers, whereas the application of low concentrations increases nodule numbers, indicating that cytokinin has both positive and negative effects on nodulation depending on the dose (Mens et al., 2018).

## Involvement of miR2111 in CLE and LRR-RLK Long-Distance Negative-Feedback Regulation of Nodulation

Evidence that miR2111 is a crucial shoot-derived factor regulating nodule numbers has been accumulating rapidly (Figure 1; Tsikou et al., 2018; Gautrat et al., 2020a; Okuma et al., 2020; Zhang et al., 2021). In *L. japonicus*, *M. truncatula*, and soybean, miR2111 targets *TML(s)* mRNA and can enhance rhizobial infection. miR2111 overexpressing transgenic plants typically exhibit a hypernodulation phenotype. In both leaves and roots, mature miR2111 accumulation and the expression of several miR2111 genes are immediately suppressed in response to rhizobial inoculation and nitrate treatment depending on shoot-acting LRR-RLKs, such as HAR1 in *L. japonicus*, SUNN in *M. truncatula*, and NARK in soybean. Therefore, miR2111 was hypothesized to have systemic effects on root nodulation control. Indeed, mature miR2111s are preferentially synthesized in leaves and the expression of its target, *TML*, occurs only in roots (Tsikou et al., 2018; Gautrat et al., 2020a; Okuma et al., 2020; Zhang et al., 2021). Intriguingly, shoot-less *L. japonicus* roots, mechanically separated from the shoot, cannot form nodules and express lower miR2111 levels (Tsikou et al., 2018). In contrast, shoot-less miR2111-overexpressing roots and *tml* roots can produce nodules, indicating that mature miR2111 production in the shoot could be necessary to control nodulation. Several experiments, such as grafting experiments of miR2111-overexpressing and downregulated transgenic lines in *L. japonicus* and petiole-feeding assays of synthetic miR2111 in soybean further demonstrated that shoot-derived miR2111 can systemically enhance nodulation by influencing *TML* mRNA levels in roots (Okuma et al., 2020; Zhang et al., 2021). Overall, the miR2111-*TML* module might be widely conserved in legumes. Although, the decrease in miR2111 with rhizobial inoculation is not completely abolished in soybean *nark* and *L. japonicus har1* mutants (Okuma et al., 2020; Zhang et al., 2021). Moreover, the induction of *TML* expression is not fully dependent on *L. japonicus* HAR1 and *M. trancatula* SUNN (Gautrat et al., 2020a; Okuma et al., 2020). A possible explanation is that LRR-RLKs, such as HAR1, SUNN, and NARK, might form receptor complexes with other proteins. For example, SUNN in *M. truncatula* was reported to interact with CLAVATA2 (CLV2), a leucine-rich repeat receptor protein, and CORYNE (CRN), a pseudokinase (Crook et al., 2016). In addition, *clv2* and *crn* mutants exhibit shoot-controlled hypernodulation phenotypes similar to that with *sunn*. Hence, these putative LRR-RLK binding partners might be individually involved in miR2111-*TML* node regulation. Another possibility is the involvement of cytokinin-related pathways in roots. Tsikou et al. (2018) showed that miR2111 suppression with rhizobial inoculation in *L. japonicus* is partially dependent on the cytokinin receptor LOTUS HISTIDINE KINASE1 (LHK1) in roots. Additionally, Miri et al. (2019) showed that *lhk1 har1*-double mutants cannot induce *TML* expression in response to rhizobial inoculation, indicating that LHK1 and HAR1 have an additive effect on regulating *TML* expression. Since LHK1 is involved in *CLE-RS3* expression induction (Miri et al., 2019), decreased production of root-derived signals might cause high miR2111 levels and low *TML* mRNA levels, which were observed in *lhk1* when rhizobia were inoculated. In addition, cytokinin application causes NIN-dependent transcriptional activation of *M. truncatula CLE13* through CYTOKININ RESPONSE1 (CRE1) cytokinin receptor, an LHK1 ortholog (Mortier et al., 2012). This suggests that CRE1 and LHK1 are likely involved in AON upstream of NIN (Figure 1). Alternatively, LHK1 might locally influence *TML* expression in roots, independent of shoot-acting receptors such as HAR1/SUNN/NARK (Figure 1).

## Root-Derived C-Terminally Encoded Peptide-Mediated Positive Regulation of Nodulation *Via* miR2111 Under Nitrogen-Deprived Conditions

Nitrogen availability is a key factor determining nodule numbers in roots. For example, under high nitrate conditions, a NIN-LIKE PROTEIN (NLP) transcription factor of *L. japonicus*, referred to as NITRATE UNRESPONSIVE SYMBIOSIS 1 (NRSYM1/NLP1), directly activates *CLE-RS2* expression and systemically inhibits nodulation through the AON pathway (Nishida et al., 2018). In contrast to AON, Gautrat et al. (2020a) showed that under limited nitrogen availability, root-derived CEP small peptides systemically enhance rhizobial infection through the LRR-RLK COMPACT ROOT ARCHITECTURE 2 (CRA2), an ortholog of CEP RECEPTOR 1 (CEPR1) in *Arabidopsis thaliana* (hereafter *Arabidopsis*; Figure 1). Compared to wild-type plants, *cra2*-mutants produce fewer nodules under nitrogen-limiting conditions, suggesting that CEP-CRA2 interaction is necessary to produce a sufficient number of nodules similar to that of wild-type plants (Huault et al., 2014). Grafting experiments of *cra2* demonstrated that CRA2 acts in the shoot to control nodulation. Intriguingly, *M. truncatula* CRA2 induces miR2111 accumulation in the shoot, thereby systemically promoting rhizobial infection by influencing *TML1* and *TML2* mRNA levels in roots in response to nitrogen deficiency (Gautrat et al., 2020a). Although the SUNN-mediated negative and CRA2-mediated positive feedback pathways recruit the same downstream factors, miR2111 and *TMLs*, to control susceptibility to rhizobia, these pathways seem to independently regulate nodulation. *Sunn cra2*-double mutants form intermediate nodule numbers between *sunn*- and *cra2*-single mutants (Laffont et al., 2019). Antagonistic regulation of nodulation by SUNN and CRA2 pathways might contribute to maintaining optimal nodule numbers to adapt to fluctuating soil nitrogen availability.

The responsiveness of miR2111 to nitrate is different between legumes and *Arabidopsis* (Liang et al., 2015; Tsikou et al., 2018; Gautrat et al., 2020a). The accumulation of miR2111 is increased by nitrate deficiency in legumes, whereas it is decreased in *Arabidopsis*. A possible explanation for this discrepancy might lie in distinctions in the shoot-derived factors downstream of the CEP-CEPR (or CRA2) module, the long-distance signaling components that inform nitrate starvation (Ohkubo et al., 2017; Gautrat et al., 2020a). In *Arabidopsis*, CEP is a long-distance signal that informs local soil nitrate deficiency on one side of roots to distant roots (where nitrate availability is relatively high), through root-derived CEP recognition by the shoot-localized LRR-RKs CEPR1 and CEPR2 (Tabata et al., 2014; Ohkubo et al., 2017). After CEP perception by CEPR1 and CEPR2, CEP DOWNSTREAM 1 (CEPD1) and CEPD2 are induced and synthesized in the shoot. CEPD1 and CEPD2 are translocated from the shoot to the roots and enhance nitrate uptake through *NRT2.1* induction, which encodes a high-affinity nitrate transporter. In contrast, in *M. truncatula*, CRA2 induces miR2111 accumulation in shoots to remove the restriction of rhizobial infection by *TML1* and *TML2* in roots (Gautrat et al., 2020a). *Medicago truncatula* CRA2 also induces a CEPD ortholog, of which the functional relevance is unknown, whereas its expression occurs both in shoots and roots (Gautrat et al., 2020a). This implies that CEP-mediated adaptation strategies to nitrogen starvation are likely regulated in a different manner between *Arabidopsis* and legumes. Although the relationship between CEPR1/2 and miR2111 in *Arabidopsis* is unknown, miR2111 recruitment by legumes in the nitrate starvation response through the CEP-CEPR module might have contributed to the gain of root nodule symbiosis in legumes.

## Adaptations to Phosphate Deficiency Through AON

Originally, miR2111 was identified as a phosphate (Pi)-responsive miRNA in *Arabidopsis* (Hsieh et al., 2009; Pant et al., 2009). Although the relationship between Pi and miR2111 in legumes is unclear, Isidra-Arellano et al. revealed that in *Phaseolus vulgaris*, nodulation inhibition induced by Pi deficiency is controlled by AON signaling components, including *P. vulgaris TML*, *RHIZOBIA-INDUCED CLE 1/2* (*RIC1/2*), and *NARK* (Figure 1; Isidra-Arellano et al., 2018, 2020). The low Pi conditions induce *RIC1*, *RIC2*, and *TML* expression, suggesting the involvement of these genes in the low-nodulation phenotype of *P. vulgaris* under Pi-deficient conditions. *RIC1* and *RIC2* induction in response to Pi deficiency is likely dependent on PHOSPHATE STARVATION RESPONSE 1 (PHR1), a master transcription factor for Pi deficiency adaptation (Rubio et al., 2001; López-Arredondo et al., 2014; Isidra-Arellano et al., 2020). The putative cis-regulatory element P1BS, a *PHR1*-binding sequence (Bustos et al., 2010), was identified in *RIC1* and *RIC2* promoters, and this putative cis-element is conserved in promoter regions of *RIC1* and *RIC2* orthologs of other legumes, implying the involvement of *PHR1* in this negative regulation of nodulation. Indeed, *RIC1* and *RIC2* expression induction by Pi deficiency is not observed in *PHR1*-silenced *P. vulgaris* hairy roots. Moreover, grafting experiments using *P. vulgaris* and soybean *nark* mutants demonstrated that this nodulation restriction under Pi deficiency is also a shoot-controlled negative regulatory system that depends on NARK (Isidra-Arellano et al., 2020). Since elevated *TML* mRNA levels in roots have been observed in response to low Pi levels, it is not surprising that miR2111 is also involved in this regulation.

The involvement of miR2111 in the regulation of symbiosis with arbuscular mycorrhizal fungi (AMF) was hypothesized (Oldroyd and Leyser, 2020). More than 70% of land plant species establish symbiotic relationships with AMF and take advantage of nutrient acquisition, especially Pi, through fungal hyphae. Since host plants must provide AMF with photosynthates to maintain AM symbiosis, they inhibit mycorrhization under high-Pi conditions. AM symbiosis is evolutionarily older than legume–*Rhizobium* symbiosis, and many of regulatory mechanisms of AM symbiosis have been co-opted to regulate legume–*Rhizobium* symbiosis (Markmann and Parniske, 2009). In *M. truncatula*, specific CLE peptides are induced in roots in response to high Pi concentrations and inhibit AMF colonization through shoot-acting SUNN receptor-like kinase (Müller et al., 2019; Karlo et al., 2020). Considering that SUNN regulates miR2111 accumulation in the AON pathway (Gautrat et al., 2020a), AMF colonization rates might also be regulated *via* miR2111 downstream of the *CLE*-*SUNN* interaction. However, if TML functions to inhibit mycorrhization, this hypothesis contains a contradiction. This is because under low-Pi conditions, the *TML* mRNA level increases, as described, while the rate of AM colonization also increases (Menge et al., 1978; Thomson et al., 1986; Breuillin et al., 2010). Therefore, unknown downstream factors of SUNN might regulate AM symbiosis other than the miR2111-*TML* module.

## Future Perspective

As described, the functional relevance and mechanism of miR2111 in legumes have become evident in recent studies (Figure 1). However, some points require further clarification. First, the tissues and cell layers of roots in which miR2111 would interact with *TML* mRNA have not been investigated. Detailed histological analysis could discern the accurate molecular mechanism of the miR2111-mediated control of *TML* and rhizobial infection. Second, how miR2111 is transported to the roots is unclear. It is also unknown if there any specific carrier for miR2111 or is just a diffusion process. Further, how miR2111 can arrive at the correct root tissue and cell layer where *TML* mRNA is produced is a fascinating question, since TML negatively regulates rhizobial infection in root hairs, as well as cortical cell division. Third, the mechanism underlying the regulation of miR2111 by LRR-RLKs such as HAR1/SUNN/NARK and CRA2 also remains unknown. Further investigation of the downstream factors of these LRR-RLKs could answer this outstanding question. Lastly, the functional relevance of miR2111 and *TML* in non-legumes remains unknown. In *Arabidopsis*, tobacco, and rapeseed, miR2111 accumulates in response to Pi deficiency (Hsieh et al., 2009; Pant et al., 2009; Buhtz et al., 2010; Huen et al., 2018). Moreover, results of *Arabidopsis* grafting experiments and deep sequencing of rapeseed phloem sap demonstrated that miR2111 has shoot-to-root mobility (Pant et al., 2009; Buhtz et al., 2010). Therefore, miR2111 in non-legumes, such as *Arabidopsis* and tobacco, could contribute to Pi-starvation adaptation systemically. Since miR2111 targets mRNA of the *TML* orthologs gene in *Arabidopsis* and tobacco, the miR2111-*TML* relationship is likely conserved in non-legumes (Huen et al., 2017, 2018). The sequences of miR2111- and miR2111-target sites in *TML* and its ortholog in legumes, *Arabidopsis*, and tobacco are well-conserved (Hsieh et al., 2009; Huen et al., 2018; Tsikou et al., 2018). Because almost all non-legumes cannot form nodules, miR2111 could have a narrower function in those plants compared with that in legumes. Indeed, *Arabidopsis* and rapeseed only have two copies of miR2111, whereas *L. japonicus*, *M. truncatula*, and soybean have seven, 15, and six copies of miR2111, respectively (Gautrat et al., 2020a; Okuma et al., 2020; Zhang et al., 2021). These differences in the duplication degree of miR2111 might correlate with its diversity in miR2111 functions. Given that many nutrient-responsive genes have been leveraged for root nodule symbiosis regulation (Oldroyd and Leyser, 2020; Roy et al., 2020), it is plausible that the miR2111-*TML* module has unknown functions related to responses to soil nutrition, such as Pi. Another possibility is that miR2111-TML might be involved in AM symbiosis control. In tomato, *clv2* mutants exhibit increased mycorrhizal colonization rates (Wang et al., 2018). Moreover, grafting experiments using tomato *clv2* demonstrated that CLV2 might act in both the shoot and roots to regulate AMF colonization (Wang et al., 2021). Since CLV2 physically interacts with shoot-acting LRR-RLK of legumes such as SUNN *via* CORYNE in *M. truncatula* (Crook et al., 2016), further investigation of the relationship between CLV2 and AM symbiosis in non-legumes is required. In particular, the elucidation of miR2111 and *TML* function may provide novel insights into how root nodule symbiosis regulatory mechanisms were acquired from pre-existing systems.

## Author Contributions

All authors listed have made a substantial, direct and intellectual contribution to the work, and approved it for publication.

### Conflict of Interest

The authors declare that the research was conducted in the absence of any commercial or financial relationships that could be construed as a potential conflict of interest.
